# Supplementary material for: Preconception health in adolescence and adulthood across generations in the UK: Findings from three British birth cohort studies
Source: PLoS One. 2024 Dec 11;19(12):e0299061. doi: 10.1371/journal.pone.0299061 (PMC11633974; doi:10.1371/journal.pone.0299061)
Supplement: S1 Table — (DOCX) [file pone.0299061.s001.docx]

**S1 Table.** Overview and definitions of preconception indicators consistently recorded in at least two sweeps at age 16/17 and/or age 25/26 years in the 1970 British Birth Cohort Study (BCS70), Next Steps and Millennium Cohort Study (MCS)

| **Preconception indicator** | **Proposed measure^a^** | **Sweeps containing proposed measure** | **Related measure** | **Sweeps containing related measure** | **Final categories** |
| --- | --- | --- | --- | --- | --- |
| ***Wider determinants of health*** | | | | | |
| Ethnicity | Percentage of women from a minority ethnic group | BCS70-16y  BCS70-26y  Next Steps-16y  Next Steps-25y |  |  | Yes (Indian, Pakistani, Bangladeshi, Asian, Chinese, Black, Mixed, other)  No (White/European) |
| Education | Percentage of women who have not completed high school education | BCS70-26y  Next Steps-25y |  |  | Yes (left school at age 16 or younger/not completed A levels)  No (left school at age 17 or older/completed A levels) |
| Employment | Percentage of women who are unemployed and seeking work | BCS70-26y  Next Steps-25y |  |  | Yes (currently unemployed and seeking work)  No (currently employed or doing something else [not looking for work]) |
| Housing | Percentage of women who do not have access to safe, comfortable, affordable housing |  | Percentage of women who are homeless | Next Steps-25y  MCS-17y | Yes (homeless, living in sheltered housing or a hostel for homeless, refuge, YMCA, etc)  No (not homeless of living in sheltered housing or a hostel for homeless, refuge, YMCA, etc) |
| Migrant health factors | Percentage of immigrant and refugee women |  | Percentage of women with language other than English usually spoken at home | BCS70-16y  MCS-17y | Yes (English + other language, other language + some English, other language only)  No (English language only) |
| ***Reproductive health and family planning*** | | | | | |
| Obstetric history | Percentage of women (who have previously been pregnant) with a previous fetal death, miscarriage or stillbirth |  | Percentage of women (who have previously been pregnant) with a previous miscarriage, termination or stillbirth | Next Steps-25y  MCS-17y | Yes (had a pregnancy resulting in stillbirth, termination and/or miscarriage)  No (had a pregnancy but not experienced stillbirth, termination or miscarriage) |
| ***Health behaviours and weight*** | | | | | |
| Dietary intake | Percentage of women not consuming a healthy diet (e.g. in line with the Eatwell Guide) |  | Percentage of women not consuming fruit daily | BC70-16y  MCS-17y | Yes (consuming fruit less than 7 days/week)  No (consuming fruit 7 days per week) |
|  |  |  | Percentage of women consuming soft drinks >1 serve/day | BC70-16y  Next Steps-25y  MCS-17y | Yes (consuming 2 or more glasses per day)  Np (not usually consuming soft drink or 1 glass per day) |
|  | Percentage of men not consuming a healthy diet (e.g. in line with the Eatwell Guide) |  | Percentage of men not consuming fruit daily | BC70-16y  MCS-17y | Yes (consuming fruit less than 7 days/week)  No (consuming fruit 7 days per week) |
|  |  |  | Percentage of men consuming soft drinks >1 serve/day | BC70-16y  Next Steps-25y  MCS-17y | Yes (consuming 2 or more glasses per day)  Np (not usually consuming soft drink or 1 glass per day) |
| Weight | Percentage of women in the underweight BMI category (<18.5 kg/m^2^) | BCS70-16y  Next Steps-25y  MCS-17y |  |  | Yes (BMI <18.5 kg/m^2^)  No (BMI ≥18.5 kg/m^2^) |
|  | Percentage of women in the overweight BMI category (25.0-29.9 kg/m^2^) | BCS70-16y  Next Steps-25y  MCS-17y |  |  | Yes (BMI 25.0-29.9 kg/m^2^)  No (BMI <25.0 or ≥30 kg/m^2^) |
|  | Percentage of women in the obese BMI category (≥30.0 kg/m^2^) | BCS70-16y  Next Steps-25y  MCS-17y |  |  | Yes (BMI (≥30.0 kg/m^2^)  No (BMI <30 kg/m^2^) |
|  | Percentage of men in the overweight BMI category (25.0-29.9 kg/m^2^) | BCS70-16y  Next Steps-25y  MCS-17y |  |  | Yes (BMI 25.0-29.9 kg/m^2^)  No (BMI <25.0 or ≥30 kg/m^2^) |
|  | Percentage of men in the obese BMI category (≥30.0 kg/m^2^) | BCS70-16y  Next Steps-25y  MCS-17y |  |  | Yes (BMI (≥30.0 kg/m^2^)  No (BMI <30 kg/m^2^) |
| Tobacco use | Percentage of women who currently smoke | BCS70-16y  BCS70-26y  Next Steps-16y  Next Steps-25y  MCS-17y |  |  | Yes (currently smoking cigarettes [past week; every day or occasionally])  No (not currently smoking cigarettes [past week]) |
|  | Percentage of men who currently smoke | BCS70-16y  BCS70-26y  Next Steps-16y  Next Steps-25y  MCS-17y |  |  | Yes (currently smoking cigarettes [past week; every day or occasionally])  No (not currently smoking cigarettes [past week]) |
| Alcohol consumption | Percentage of women who consume alcohol | BCS70-16y  BCS70-26y  Next Steps-16y  Next Steps-25y  MCS-17y |  |  | Yes (consuming any alcohol; from every day to on special occasions only)  No (none/never) |
|  | Percentage of men who consume excessive amounts of alcohol (>14 units per  week) |  | Percentage of men who consume any alcohol | BCS70-16y  BCS70-26y  Next Steps-16y  Next Steps-25y  MCS-17y | Yes (consuming any alcohol; from every day to on special occasions only)  No (none)  (not possible to quantify units per week) |
| ***Physical health conditions*** | | | | | |
| Epilepsy | Percentage of women with (uncontrolled or unreviewed) epilepsy |  | Percentage of women with epilepsy | BCS70-26y  MCS-17y | Yes (had epilepsy in past 12 months)  No |
|  | Percentage of men with epilepsy | BCS70-26y  MCS-17y |  |  | Yes (had epilepsy in past 12 months)  No |
| Diabetes mellitus | Percentage of women with (uncontrolled or unreviewed) type 1 or type 2 diabetes |  | Percentage of women with type 1 or type 2 diabetes | BCS70-26y  Next Steps-25y  MCS-17y | Yes (had diabetes in past 12 months)  No |
|  | Percentage of men with diabetes | BCS70-26y  Next Steps-25y  MCS-17y |  |  | Yes (had diabetes in past 12 months)  No |
| Asthma | Percentage of women with (uncontrolled or unreviewed) asthma |  | Percentage of women with asthma | BCS70-16y  BCS70-26y | Yes (had asthma in past 12 months)  No |
| Cancer | Percentage of women with (prior) cancer (treatment) | BCS70-26y  Next Steps-25y |  |  | Yes (had cancer since age 16)  No |

^a^ Proposed preconception indicator measures based on Schoenaker et al., 2022
