# Supplementary material for: Preconception health in adolescence and adulthood across generations in the UK: Findings from three British birth cohort studies
Source: PLoS One. 2024 Dec 11;19(12):e0299061. doi: 10.1371/journal.pone.0299061 (PMC11633974; doi:10.1371/journal.pone.0299061)
Supplement: S2 Table — (DOCX) [file pone.0299061.s002.docx]

**S2 Table.** List of preconception indicators not consistently recorded in at least two sweeps at age 16/17 and/or age 25/26 years in the 1970 British Birth Cohort Study (BCS70), Next Steps and Millennium Cohort Study (MCS)^a^

| **Wider determinants of health**   - Financial security - Deprivation - Complex social factors - Adverse childhood experiences |
| --- |
| **Health care**   - Preconception assessment and care - Routine general practitioner (GP) check-up - Routine dental appointment |
| **Emotional and social care and support**   - Domestic abuse (physical and emotional) - Support network / social support |
| **Reproductive health and family planning**   - Pregnancy intention - Maternal age - Paternal age - Interpregnancy interval - Previous breastfeeding experiences - Fertility problems - Contraception - Assisted reproductive technology (ART) |
| **Health behaviours and weight**   - Folic acid supplementation - Other vitamin supplementation - Vitamin deficiency - Physical activity - Eating disorder - Second-hand smoke exposure - Substance use |
| **Environmental exposures**   - Toxic or hazardous substances or exposures |
| **Cervical screening**   - Cervical screening |
| **Immunisations and infections**   - Immunisation - Sexually transmitted diseases |
| **Mental health conditions**   - Mental health condition - Previous antenatal or postnatal mental illness - Severe mental health condition |
| **Physical health conditions**   - Polycystic ovary syndrome (PCOS) - Endometriosis - Thyroid disease - Chronic hypertension - Cardiovascular disease - Previous thromboembolism - Renal disease - Rheumatological conditions - Inflammatory bowel disease - Sickle-cell disease or thalassaemia - Hepatitis B - Lupus - Phenylketonuria (PKU) - Developmental disability - Female genital mutilation - Pelvic floor dysfunction |
| **Medication use**   - Overall (any) medication use - Medication not recommended when planning pregnancy |
| **Genetic risk**   - Personal or family history of a generic disorder - Previous pregnancy affected by an inherited genetic disorder - Family history of diabetes |

^a^ Preconception indicators as outlined in Schoenaker et al., 2022
