# Supplementary material for: Preconception health in adolescence and adulthood across generations in the UK: Findings from three British birth cohort studies
Source: PLoS One. 2024 Dec 11;19(12):e0299061. doi: 10.1371/journal.pone.0299061 (PMC11633974; doi:10.1371/journal.pone.0299061)
Supplement: Attachment — Submitted filename: Response to reviewers.docx [file pone.0299061.s003.docx]

**Response to reviewers' comments:**

**Reviewer: 1**

The authors present an informative and timely investigation into the current state and trends in preconception health in the UK. In my view, descriptive studies like this, reporting on general population samples, are very important in guiding policymakers, for raising further, more complex research questions, and informing professionals in many fields. I think there is a strong rationale (intellectual, moral, and economic one) for improving children’s well-being by focusing on ‘upstream’ factors and by improving the health and well-being of the families they are born to. As mentioned by the authors, it is not only the children that benefit, and improvement in preconception health will benefit at least two generations. The key strength of the study has to do with using population-based studies, as other types of data sources might focus only on women or pregnant individuals, for example.

| **Comment 1:** The introduction section makes a strong case for the study. It highlights the impact that improving preconception health can have on multiple generations, and the authors seem to cover previous approaches to describing preconception health in the UK well. However, I wonder what the authors' rationale is for not describing findings from other countries? There are, of course, important differences in these phenomena and service systems even between European countries, but maybe there would be some previous findings from other countries that would be relevant for the current study? I appreciate that the authors might have an excellent rationale for adopting a UK-focused approach, but the rationale for that is not completely clear to me after reading the introduction section. I also think that the results will be able to inform professionals from areas outside of the UK. | Thank you for your feedback. Our rationale for adopting a UK-focused approach is rooted in the availability and depth of data from the British birth cohort studies, but we acknowledge the importance of considering findings from other countries in informing UK policy. The British birth cohort studies are unique in their longitudinal design, tracking individuals across their reproductive years and enabling comparison between different generational cohorts. While these studies provide insights into preconception health specifically within the UK context, this study’s findings possess transferable value for shaping policy in similar countries. We have updated the following sentence in the conclusion accordingly:  *“These findings serve as essential benchmarks for designing targeted interventions and shaping public health policies in the UK and other similar countries to address obesity, improve dietary patterns, reduce alcohol and tobacco consumption further, and ensure the overall well-being of the preconception population.”* (Lines 417-420) |
| --- | --- |
| **Comment 2:** I think the data in Table 1 would be best presented visually, or at least in a slightly different way. Although the authors have reported all relevant data in the table, currently it is somewhat difficult to read and to assess the overlap between data collection waves and the assessment ages across studies. | We have now visually presented the data collection sweeps during the reproductive years, showing the alignment of age at the time of data collection across the three cohorts (Figure 1, page 5). |
| **Comment 3:** It seems to me that all three survey studies included individuals born in the UK. If this is the case, the implications of this need to be discussed clearly in the discussion section. | This is correct, all individuals included in the study were born in the UK. To make this clearer, we have added to both the discussion section's first paragraph and the conclusion that these data are from the UK (Lines 270 & 412). |
| **Comment 4:** I think all in all the analysis approach is clear, and I appreciate the simplicity of it. If possible, I would prefer statistical testing that would provide estimates of the magnitude of the effect size, but of course, the magnitude of observed associations can be communicated in other ways too. | Thank you for your feedback. Statistical testing to provide estimates of the magnitude of effect sizes is highly dependent on the sample size. We therefore prefer to describe the results and interpret the public health relevance, based on actual differences.  For example, we communicate the magnitude of the rise in obesity as: *“The prevalence of overweight and obesity displayed an upward trajectory over time, with adolescents in the MCS-17y cohort being around five times (females) to six times (males) more likely to live with obesity when compared to their counterparts in the BCS70-16y cohort.”* (Lines 275-278) |
| **Comment 5:** I think it would be important to report more information on the survey samples (e.g. missing data) for each survey in more detail than it has been done now. | We have addressed this suggestion by adding more detailed information about the samples for each survey in the results section:  *“At age 16, the maximum sample sizes were 11,615 for BCS70, 12,439 for Next Steps, and 10,757 for MCS-17y. At age 26, BCS70 had a maximum sample size of 9,003, and at age 25, Next Steps had 7,707 participants. These numbers reflect the loss to follow-up, given the initial sample sizes of around 17,000, 16,000, and 19,000 for BCS70, Next Steps, and MCS-17y, respectively.”* (Lines 148-152) |
| **Comment 6:** I find it somewhat hard to follow the results as they are being reported currently in the text. This might only be a matter of personal preferences, but I would consider doing some restructuring, and not reporting on each indicator separately. One thing that might simplify the results section would be that the descriptions of how the indicators were defined, and when information on each was available, would be described in a separate subsection of the results section, potentially in a table. | Thank you for your feedback. We've worked on enhancing the clarity of the results section by trimming unnecessary details to streamline the presentation. We've opted to only list specific sweeps rather than providing information on how many sweeps contained the data. However, we maintain that presenting each indicator separately offers a more comprehensive understanding of the findings. |
| **Comment 7:** For some indicators (for example, for Ethnicity), it is not completely clear to me whether there was no significant difference found between ages/birth cohorts, or whether no statistical comparison was conducted. The reporting style could be made more clear. Otherwise the manuscript is very clearly written. | Thank you for your feedback. A footnote has been added to specify that p-values are provided for variables with two or more available data points for each indicator (Line 177).  In Table 2 (now Table 1), p-values comparing cohorts at age 16/17 and 26/27 are provided, where applicable. For ethnicity, a significant difference in the proportion of individuals from an ethnic minority (p=<0.0001) was observed between BCS70 and Next Steps at both age 16/17 and age 26/27. However, data for MCS were not available for comparison. |
| **Comment 8:** I very much agree with the authors about the need for ongoing monitoring of health indicators from a public health point of view. I think the authors could describe in the discussion section what they think would be the best approach to this going forward, and what their recommendations are so that an even better monitoring can be done in the future. | We recommend standardised questions and data collection across regular sweeps and birth cohorts to improve ongoing monitoring (lines 399-402).  We have added that *“When compared with routinely collected health(care) data, survey data from the British birth cohorts provide unique new information on important preconception indicators such as dietary intake, alcohol consumption, education and housing. Collectively, this could be used alongside preconception indicators from other data sources to track progress...*” (Lines 402-406). |
| **Comment 9:** Mental disorders are very much associated with one’s somatic health, health behaviors, ability to study and work, and importantly in the context of preconception health, they are associated with things such as the ability and willingness to take care of children, medications used before and during pregnancy, and breastfeeding. I think mental health is a very important dimension that should be considered when assessing preconception health. I appreciate that including these in the current study was not possible because of the limitations of the datasets, but I do think mentioning these factors would be important in the discussion section. Again, I don’t think this is necessarily a weakness of the current study, but given that the aim is to improve preconception health and our understanding of the current state of it, I think the importance of also assessing mental disorders could be mentioned here too. | Thank you for emphasising the importance of mental health in assessing preconception health. We have now listed the absence of consistent mental health data as a limitation of the datasets in the discussion section:  *“The inconsistent assessment of mental health across cohorts represents a notable limitation of the datasets. Specifically, the lack of data pertaining to mental health disorders and associated prescribed medications presents a significant drawback, given the potential for teratogenicity.”* (Lines 359-362) |

**Reviewer: 2**

Thanks for the opportunity to review this interesting paper which is really well written. I have a couple of questions and suggestions I would be grateful if you could clarify please.

| **Comment 1:** You reported using cohort sweeps with comparable ages of participants of 16-17 and 25-26, but this meant excluding the most recent data from MCS, where the 2023 sweep captured data from 23 year olds. I would think 23 year olds are close enough in age to 25-26 year olds to include these most up to date data which would be informative for current policy. Please could you explain why you excluded the age 23 MCS sweep? (Presumably this post-school age MCS sweep also includes the education indicator too? And including it would enable MCS to be included in Table 3?) Ideally I would recommend that the data from MCS age 23 sweep be included in the paper which is why I have recommended a major revision, my other comments are minor. | Thank you for your comment and request for clarification. We looked at data from sweeps where participants were of the same age across the cohorts, within a 1-year age range to maximise the use multiple cohorts (see updated figure 1). The age 23 MCS sweep was therefore not included.  We looked at age 16/17 (adolescence) and 25/26 years (early adulthood). Age 23 would fall within the definition of adolescence (10-24 years; <https://doi.org/10.1016/S2352-4642(18)30022-1>). Including the age 23 MCS sweep with BCS70-26y and Next Steps-25y may therefore not be appropriate.  We believe that further work, beyond the scope of our paper, is needed to continue to monitor recent trends in preconception health across the life course to inform policy. This has now been emphasised more in the discussions *“The findings also call for the use of standardised questions and data collection on key public health measures across regular and more recent sweeps and birth cohorts to support ongoing monitoring of preconception health indicators and inform policy.”* (Lines 399-402) |
| --- | --- |
| **Comment 2:** I think when referring to differences between cohorts it would be clearer to say differences across cohorts instead of across sweeps. For example at line 220, I think "Among female participants, the proportion of those aged 16/17 who smoked was significantly different across sweeps" should read different across cohorts. This occurs later too e.g. line 235. The phrase different across sweeps could then be used for change within cohorts between different sweeps. | Thank you for your suggestion. We have reviewed the text and updated it accordingly to make the distinction between sweeps and cohorts clearer and correct the mistakes you point out (Lines 225 and 239). |
| **Comment 3:** The sentence at line 327 is not entirely clearly worded: Moreover, increases of 33-44% of pregnancies complicated by type 1 diabetes and 90-111% of pregnancies complicated by type 2 diabetes have been reported in Northern Europe (30,31). | Thank you for your feedback. This sentence has been updated to:  *“Moreover, Northern Europe has reported increases of 33-44% in pregnancies complicated by type 1 diabetes and increases of 90-111% in pregnancies complicated by type 2 diabetes have been reported in Northern Europe (30,31).”* (Lines 331 – 333) |
| **Comment 4:** It would be helpful to understand the exact questions that were asked for indicators where you propose differences in interpretation could have influenced the results (e,.g categorisation of termination of pregnancy as a 'complication' or not. | Thank you for your suggestion. We have added detail on the question asked for this indicator where differences in interpretation may have influenced the results:  *“Specifically, participants were asked whether those who had been pregnant had experienced a termination, miscarriage, or stillbirth. It is possible that adolescents in MCS-17y had recent experience of pregnancy termination, while adult women in Next Steps-25y may not have reported these as such, especially in cases of past unintended teenage pregnancy.”* (Lines 382 – 385) |
