# Supplementary material for: Preconception health in adolescence and adulthood across generations in the UK: Findings from three British birth cohort studies
Source: PLoS One. 2024 Dec 11;19(12):e0299061. doi: 10.1371/journal.pone.0299061 (PMC11633974; doi:10.1371/journal.pone.0299061)
Supplement: Attachment — Submitted filename: Response to reviewers.docx [file pone.0299061.s004.docx]

**Response to reviewers' comments:**

| **Comment 1:** As suggested by Reviewer 1, please describe in the Introduction-section, with references, what is known about this topic in other high-income countries. I appreciate the study is about the UK, but it is important to provide a clear context for the investigation, explicitly explaining what this study adds to what we already know about this topic. | Thank you for your feedback. We have added a paragraph to the Introduction section, providing an overview of what is known about this topic in other high-income countries, with relevant references (lines 39 – 53). We have also included this evidence for comparison in the discussion (lines 307-310 and 329-332. |
| --- | --- |
| **Comment 2:** As suggested by Reviewer 2, please add analyses using data from the MCS age 23 years sweep. You cite a paper proposing that adolescence these days extends to age 24, but life course epidemiology strongly suggests that health and outcomes in early adulthood are comparable in the early to mid-20s. Further, given the limited sample sizes, particularly on some indicators, adding MSC data from the age 23 sweep would increase the value of the study considerably. | The MCS age 23 years sweep is currently ongoing, and data won’t be available until the end of 2025. We are therefore not able to add analysis using this sweep as part of the current paper. This has now been clarified in the methods section (lines 114-116). Please find more information here: <https://cls.ucl.ac.uk/cls-studies/millennium-cohort-study/mcs-age-23-sweep/> |
| **Comment 3:** As a general comment, the interpretation of the findings needs to be toned down throughout the manuscript. In multiple places the authors write about their findings revealing or showing patterns. Given the observational nature of the studies included, the modest sample sizes, attrition and missing data, these types of statements on the findings are too strong. At the most, the evidence from these studies can suggest (not reveal) associations. | Thanks for your feedback. We have carefully reviewed the manuscript and replaced terms such as 'shows' and 'reveals' with 'observed' and 'noted' throughout the discussion to better reflect the observational nature of the studies and avoid overstating the findings. |
| **Comment 4:** The authors provide the total numbers of participants in each study in the abstract, but the numbers of individuals included in the analyses were, in fact, considerably smaller. The abstract should be revised to give the reader a realistic idea of study sizes, as this has important implications to the interpretation of the findings in terms of generalisability and analytical power. | Thank you for your feedback. We have revised the abstract and added the sentence: ***'However, data for these indicators were not consistently available for all cohort members’***, to address concerns about generalisability and analytical power. |
